# Relationship between sleep variables and interoceptive awareness in daytime workers

**DOI:** 10.1371/journal.pone.0319076

**Published:** 2025-03-12

**Authors:** Shoichi Asaoka, Ryuichiro Yamamoto, Kenta Nozoe, Ritsuko Nishimura

**Affiliations:** 1 Sleep Research Institute, Edogawa University, Nagareyama, Japan; 2 Department of Psychology and Humanities, Edogawa University, Nagareyama, Japan; Sapienza University of Rome: Universita degli Studi di Roma La Sapienza, ITALY

## Abstract

Interoception refers to the sensation of internal and physiological bodily states, such as heart rate, and contributes to the maintenance of bodily internal homeostasis. Some studies showed that interoceptive awareness is related to experiencing nightmares and subjective sleep quality. Similarly to the perception of heart rate variability, sleepiness is thought to be mainly evoked by homeostatic processes and is based on the awareness and recognition of internal body signals. However, the relationship between subjective excessive daytime sleepiness and interoceptive awareness has not been addressed. Therefore, this study examined the relationship between interoceptive awareness and multiple sleep variables including subjective excessive sleepiness in daytime workers. A web questionnaire survey was conducted targeting daytime workers in Japan, and data from 461 participants were used for analyses. Multiple regression analyses showed weak but significant relationships between subjective excessive daytime sleepiness, insomnia symptoms, nightmare distress, and dream frequency and the components of interoception awareness measured by the Multidimensional Assessment of Interoceptive Awareness. However, no components of interoceptive awareness were related to workday sleep loss or social jetlag of day workers. The results of this study suggest that subjective sleepiness, in addition to nightmare distress and sleep quality, is associated with interoceptive awareness. To the best of our knowledge, this study is the first to analyze the relationship between subjective daytime excessive sleepiness and interoceptive awareness. Further investigation of this relationship is expected to lead to a better understanding of sleep disorders and to elucidate individual differences in the accuracy of subjective assessments of sleepiness.

## Introduction

Interoception refers to the sensation of internal and physiological bodily states, such as heart rate, and contributes to the maintenance of bodily internal homeostasis [1]. Interoception has received considerable attention, and many studies have explored the relationships between interoception, emotional and motivational processes, and mental health [2].

Studies exploring interoception and sleep variables are limited. Wei and Van Someren [3] asserted the relationship between sleep and interoception based on a review of studies, which suggested that sleep deprivation enhances self-reported pain and decreases the pain threshold [4]. As an empirical study, Arora et al. [5] explored the relationship between subjective sleep quality, measured using the Pittsburgh Sleep Quality Index (PSQI) [6], and multiple dimensions of interoceptive awareness. Bynum and Brindle [7] also explored the relationship between sleep quality and interoceptive attention and accuracy with self-rating scales. They suggested a relationship between poor sleep and greater interoceptive attention. In addition, Ewing et al. [8] assessed interoception in healthy controls and participants with depression and/or anxiety using a task for measuring interoceptive performance and showed that poor sleep was associated with lower interoceptive performance accuracy and higher self-reported interoceptive sensibility in patients with depression and/or anxiety.

In addition, some research has explored the relationship between dreams, including nightmares, and interoception. The existence of pain in dreams was reported in previous studies [9,10], and the relationship between nightmare distress and interoceptive awareness with a self-rating questionnaire has also been reported [11]. Additionally, the amplitude of heartbeat-evoked potential, which is the cortical potential of time-locked to heartbeat and the psychophysiological index of interoception, is higher during rapid eye movement (REM) sleep relative to non-REM sleep [12], and its amplitude during REM sleep is higher in participants with nightmare disorders [13].

These results suggest that sleep variables, especially regarding sleep quality and nightmares, are associated with interoception. However, the relationship between interoception and other sleep-related variables such as subjective trait sleepiness and chronotype, including social jetlag (SJL; the index of the mismatch between an individual’s internal biological clock and work schedule), has rarely been examined.

Additionally, several studies have shown that sleep deprivation affects the insular cortex function [14], which is thought to play an important role in interoception [15]. Considering these issues, there might be a significant relationship between sleep loss and interoceptive awareness. Since sleepiness, as well as heart rate, is thought to be mainly evoked by homeostatic processes and is based on the awareness and recognition of internal body signals, exploring the relationship between subjective sleepiness and interoceptive awareness may improve our understanding of individual differences in the accuracy of subjective assessments of sleepiness.

Therefore, this study aimed to examine the relationship between interoceptive awareness and multiple sleep variables, mainly focused on subjective daytime sleepiness and background factors such as insufficient sleep, chronotype, sleep quality, and SJL, as well as dream experiences in daytime workers.

## Materials and methods

### Procedure and participants

All data were collected online between January 24th and 26th, 2024. The participants were recruited through Cross Marketing, Inc. (Tokyo, Japan), a web research company. We requested employed people aged 18–59 years (excluding part-time workers) registered as the company’s monitors to answer questions online. The conditions for participation were as follows: having a daytime job, not engaging in shift work, not having major health problems, and not currently attending hospital. The participants received an honorarium of web service points equivalent to JPY 30 after participating.

We examined the associations between each Multidimensional Assessment of Interoceptive Awareness (MAIA) subscale score and sleep variables using multiple regression analysis, with sleep variables as explanatory variables. In general, the number of participants required for multiple regression is approximately 10–30 times greater than the number of explanatory variables [16]. Therefore, considering the dropout rate due to unreliable answers, the target sample size was set as 300 men and 300 women before the start of the survey. We stopped the survey when a sufficient amount of data (N =  300) from participants meeting the above criteria in each gender group were collected. Finally, we obtained data from 300 men and 300 women. Their mean age was 38.49 years (standard deviation [SD] =  11.02). The study procedure was registered on the Open Science Framework platform (https://doi.org/10.17605/OSF.IO/FQUTX).

### Ethical consideration

The Research Ethics Committee of Edogawa University approved the research protocol before data acquisition (approval No. R05-012A). All procedures involving human participants performed in this study were in accordance with the ethical standards of the institutional and/or national research committee and with the 1964 Helsinki Declaration and its later amendments or comparable ethical standards. Informed consent was obtained from all the participants on the first page of web-based questionnaire.

### Questionnaire

Primary attributes (demographic variables), including age, gender, residence (prefecture), occupation, marital status, number of children, health problems, and shift work engagement were collected. We used the Japanese version of the MAIA [17] (MAIA-J [18]). The reliability (α =  .67– .88) and validity of this scale were confirmed by Shoji et al. [18]. It has six subscales: “Noticing”, “Not distracting”, “Attention regulation”, “Emotional awareness”, “Body listening”, and “Trusting”. “Noticing” comprises five items and represents the awareness of uncomfortable, comfortable, and neutral body sensations. “Not distracting” includes three items, and its score represents the tendency not to ignore or distract oneself from bodily sensation of discomfort and pain. “Attention regulation” contains seven items describing the ability to sustain and control attention to body sensation. “Emotional awareness” includes three items describing the awareness of the connection between body sensations and emotional states. “Body listening” comprises four items and represents active listening to the body for insight. “Trusting” includes three items and represents the experience of one’s body as safe and trustworthy. Participants were asked to answer each item on a 6-point Likert scale (0 to 5), and the scores of the items corresponding to each subscale were averaged, which was taken as the subscale score. Therefore, the score range of each subscale is 0–5, with higher scores indicating higher interoceptive bodily awareness.

The Japanese version of the Ultra-Short version of the Munich ChronoType Questionnaire (µMCTQ) [19,20], the Athens Insomnia Scale (AIS) [21], the Epworth Sleepiness Scale (ESS) [22,23], the Nightmare Distress Questionnaire (NDQ) [24,25], and questions regarding dream and nightmare frequencies [26] were used to measure the multidimensional daily sleep characteristics of daytime workers.

The µMCTQ is a 6-item questionnaire for assessing chronotype and was developed and confirmed for validity by Ghotbi et al. [20]. On the µMCTQ, participants are asked about the working day per week and sleep onset time (hh:mm) and end time (hh:mm) on workdays and free days, respectively. Based on sleep onset time and end time, sleep durations on workdays and on free days are calculated. Additionally, from the response to this scale, according to the method of the previous studies [19,20], sleep-corrected midsleep on free days (MSFsc) is calculated and used as a chronotype index (later time of MSFsc means eveningness chronotype), sleep loss across the week (SLOSSweek) is used as an insufficient sleep index, and SJL calculated as the absolute difference of the midpoint of sleep on free days and that on weekdays is used as an index of mismatch between an individual’s internal biological clock and work schedule.

The reliability (α = .88) and validity of the Japanese version of AIS were confirmed by Okajima et al. [21], and the total score (score range: 0–24) of eight items is used as an index of sleep quality. A higher score on this scale means more severe insomnia.

The reliability (α = .85) and validity of the Japanese version of ESS were confirmed in a previous study [23]. The total score of eight questions (score range: 0–24) is used as an index of daytime sleepiness, and a higher score means more severe daytime sleepiness.

The NDQ is a scale consisting of 13 items for assessing the distress experienced from nightmares. The reliability (α = .84) of the total score (score range: 13–65) and validity of the Japanese version were confirmed by Okada and Matsuda [25]. The total score was used as an index of nightmare-related distress in this study.

Dream and nightmare frequencies were asked separately using the items of “How often have you recalled your dreams recently (in the past several months)?” and “How often have you recalled your nightmare recently (in the past several months)?” respectively, according to Schredl et al. [26], and participants selected one of seven options (1: never – 7: almost every morning) for dream and nightmare frequency.

### Statistical analysis

Participants who did not meet the participation criteria described in the above data collection procedures (e.g., the data of the participants who answered “yes” to the question of “Do you currently have any chronic illnesses or conditions under treatment that require regular attending hospital?”) were excluded. Moreover, Cross-Marketing Inc. excluded incorrect responses with their original criteria based on response times (mean response time was less than 1 s per question), and through this process about 2% of respondents were eliminated from the data analysis. The web form was set to ask participants to make sure that their answer is right when their answer for the question in the µMCTQ about the workday sleep onset time was between 6:00 AM and 6:00 PM and/or when the workday sleep end time was between 0:00 PM (noon) and 0:00 AM (midnight). Even after this procedure, if their answer to at least one of these questions was in the above time window, their data were excluded from the analyses because their sleep–wake patterns did not match those of daytime workers. At the first step of this screening process, the data of 56 participants reporting their sleep onset time on workday between 6:00 AM and 6:00 PM were eliminated. At the second step, those of nine participants who reported their sleep end time during the afternoon on a workday were eliminated. Thereafter, according to the criteria of our previous study [27], the data of 16 participants whose calculated sleep duration on weekdays were not between 3 and 16 h were removed. Lastly, the data of 58 participants with sleep durations on free days that were not between 3 and 16 h were eliminated. Through this screening process, data from a total of 139 participants were excluded from the analysis.

In this study, a correlation matrix was used to explore the association between the MAIA score, sleep variables, and age. Stepwise multiple linear regression analyses were conducted using each MAIA subscale score as target variables, and 10 variables (MSFsc, SLOSSweek, and SJL from µMCTQ, AIS score, ESS score, dream frequency, nightmare frequency, NDQ score, and age) as explanatory variables. These variables were standardized (z) before entering the equation. Participants’ gender was entered into each equation as a dummy variable (male =  0, female =  1). All data analysis was conducted using R version 4.3 (R Foundation, Vienna, Austria).

## Results

### Summary of the participants whose data were used for analyses

Through the data screening mentioned above, data from 461 participants were used for analysis in this study. Their mean age was 38.35 (SD 10.72) years. There were 131 (28%) participants aged less than 30 years, 135 (29%) in their 30s, 108 (23%) in their 40s, and 87 (19%) in their 50s (Table 1).

**Table 1:** Summary of the participants whose data were used for analyses.

Characteristic/Variables	
Demographic	
Gender, % (n)	
Male	48.4 (223)
Female	51.6 (238)
Married, % (n)	
Yes	54.2 (250)
No	45.8 (211)
Children, % (n)	
Children living together	29.9 (138)
Children living apart	4.1 (19)
No children	65.9 (304)
µMCTQ*	
Sleep onset time, mean (SD)	
Workday	23:44 (80)
Free day	00:10 (98)
Sleep end time, mean (SD)	
Workday	06:25 (68)
Free day	08:02 (109)
Sleep duration in min, mean (SD)	
Workday	400 (70)
Free day	473 (92)
SLOSSweek in min, mean (SD)	107 (109)
MSFsc, mean (SD)	03:39 (88)
SJL in min, mean (SD)	64 (57)
AIS, mean (SD)	5.7 (4.8)
ESS, mean (SD)	7.7 (5.9)
Dream frequency, mean (SD)	3.4 (2.0)
Nightmare frequency, mean (SD)	2.3 (1.5)
NDQ, mean (SD)	23.1 (9.1)

µMCQ, Ultra-Short version of the Munich ChronoType Questionnaire; SLOSSweek, sleep loss across the week; MSFsc, sleep-corrected midsleep on free days; SJL, social jetlag; AIS, Athens Insomnia Scale; ESS, Epworth Sleepiness Scale; NDQ, Nightmare Distress Questionnaire; SD, standard deviation.

*SD represented in minutes.

### Participants’ sleep variables and interoception awareness

The sleep parameters of the participants are shown in Table 1. A paired t-test showed that sleep onset time (t (460) = 9.868, p < 0.001) and sleep end time (t (460) = 23.787, p < 0.001) were delayed, and sleep duration was longer (t (460) = 18.248, p < 0.001) on free days than on workdays. The percentage of participants with excessive daytime sleepiness (ESS ≥ 11) was 29.7% (n = 137), and that of participants with possible insomnia (AIS ≥ 6) was 46.6% (n = 215).

The mean of each subscale of the MAIA was 2.342 (SD 1.101) for “Attention regulation”, 2.311 (SD 1.150) for “Body listening”, 2.258 (SD 1.057) for “Noticing”, 2.377 (SD 1.171) for “Emotional awareness”, 2.432 (SD 1.158) for “Trusting”, and 3.954 (SD 1.184) for “Not distracting”.

### Relationship between sleep variables and interoception awareness

Before exploring the relationship between sleep variables and interoceptive awareness using multiple linear regression analyses, Pearson’s correlation coefficient was calculated between the variables (Table 2). The results of the multiple regression analyses are shown in Table 3. No variables had significant standard partial regression coefficients (beta) for “Trusting” or “Body listening” MAIA subscales. These equations were significant for other target variables. The ESS score was significantly positively related to the “Noticing” subscale. The AIS, ESS, and NDQ scores were significantly negatively related to the “Not distracting” subscale, while dream frequency was positively related. Age and ESS score were significantly positively related with “Attention regulation”. Only the NDQ score was significantly positively related with “Emotional awareness”. The results of the Kolmogorov–Smirnov test showed the normality of the residuals (all p >  0.05) of the three significant regression models.

**Table 2:** Correlation coefficients between variables.

	Noticing		Not Distracting		Attention Regulation		Emotional Awareness		Body Listening		Trusting		Age		MSFsc		SLOSS week		SJL		AIS		ESS		Dream Frequency		Nightmare Frequency		NDQ	
MAIA																														
Noticing			-.58	**	.73	**	.75	**	.68	**	.61	**	-.04		.03		.06		.09	^†^	.20	**	.21	**	.05		.15	**	.17	**
Not Distracting					-.33	**	-.35	**	-.28	**	-.23	**	.09	*	-.03		-.08	^†^	-.11	*	-.34	**	-.27	**	-.07		-.24	**	-.30	**
Attention Regulation							.77	**	.79	**	.76	**	.08	^†^	-.04		.01		.03		.05		.10	*	.04		.04		.05	
Emotional Awareness									.81	**	.77	**	-.01		.03		.04		.12	*	.09	^†^	.14	**	.08		.11	*	.13	**
Body Listening											.86	**	.00		-.02		.00		.04		.05		.08		.04		.05		.06	
Trusting													.03		-.01		.01		.04		.00		.06		.01		.03		.03	
Demographic																														
Age															-.18	**	.03		-.14	**	.03		-.08	^†^	.00		-.11	*	-.18	**
Sleep variables																														
MSFsc																	.10	*	.57	**	.05		.11	*	-.04		.03		-.01	
SLOSSweek																			.52	**	.12	*	.19	**	-.05		.03		.00	
SJL																					.10	*	.23	**	.05		.04		.06	
AIS																							.43	**	.28	**	.44	**	.54	**
ESS																									.17	**	.22	**	.29	**
Dream Frequency																											.52	**	.29	**
Nightmare Frequency																													.61	**
NDQ																														

The variables are Pearson’s correlation coefficients (r). MAIA, Multidimensional Assessment of Interoceptive Awareness; SLOSSweek, sleep loss across the week; MSFsc, sleep-corrected midsleep on free days; SJL, social jetlag; AIS, Athens Insomnia Scale; ESS, Epworth Sleepiness Scale; NDQ, Nightmare Distress Questionnaire. **p <  0.01, *p <  0.05,^†^p <  0.10.

**Table 3:** The results of multiple regression analyses targeting to MAIA subscale scores using the data of all participants.

	Target variables											
Explanatory variables	Noticing		Not distracting		Attention regulation		Emotional awareness		Body listening		Trusting	
Age					0.09	*						
Gender												
SLOSSweek												
MSFsc												
SJL							0.09					
AIS	0.10		-0.19	***								
ESS	0.15	**	-0.15	**	0.11	*	0.09		0.08			
Dream frequency			0.10	*								
Nightmare frequency	0.08		-0.10									
NDQ			-0.12	*			0.10	*				
Adj R^2^	0.06		0.15		0.01		0.03		0.00		–	
F	10.47	***	17.21	***	4.46	*	5.87	***	2.65		–	

n =  461. The values in the rows for explanatory variables are standard partial regression coefficients. MAIA, Multidimensional Assessment of Interoceptive Awareness; SLOSSweek, sleep loss across the week; MSFsc, sleep-corrected midsleep on free days; SJL, social jetlag; AIS, Athens Insomnia Scale; ESS, Epworth Sleepiness Scale; NDQ, Nightmare Distress Questionnaire. ***p <  0.001, **p <  0.01, * p <  0.05.

As an exploratory analysis, we performed the same linear multiple regressions only with data from participants (n =  196) without excessive daytime sleepiness (ESS <  11) or insomnia symptoms (AIS <  6). In the results of this analyses (Table 4), ESS scores showed significant associations with the scores of “Emotional awareness”, “Body listening”, and “Trusting”. Only age showed a significant relationship with “Not distracting”. However, other sleep variables did not show any significant relationship with MAIA subscale scores.

**Table 4:** The results of multiple regression analyses targeting to MAIA subscale scores using only the data of the participants without sleep problems.

	Target variables											
Explanatory variables	Noticing		Not distracting		Attention regulation		Emotional awareness		Body listening		Trusting	
Age	-0.11		0.14	*								
Gender												
SLOSSweek												
MSFsc												
SJL												
AIS	0.12											
ESS	0.11		-0.10		0.13		0.22	*	0.15	*	0.17	*
Dream frequency			0.14									
Nightmare frequency			-0.11									
NDQ												
Adj R^2^	0.03		0.03		0.01		0.05		0.02		0.02	
F	2.91	*	2.46	*	3.34		10.44	**	4.54	*	5.63	*

n =  196. The values in the rows for explanatory variables are standard partial regression coefficients. MAIA, Multidimensional Assessment of Interoceptive Awareness; SLOSSweek, sleep loss across the week; MSFsc, sleep-corrected midsleep on free days; SJL, social jetlag; AIS, Athens Insomnia Scale; ESS, Epworth Sleepiness Scale; NDQ, Nightmare Distress Questionnaire. ***p <  0.001, **p <  0.01, *p <  0.05.

## Discussion

This study explored the relationship between sleep variables, especially focusing on subjective daytime sleepiness and its related factors, and subjective interoceptive awareness. We conducted a web-based survey on daytime workers. Their sleep patterns were delayed and their sleep duration was longer on free days than on workdays, which means that the daytime workers who participated in this study had sleep loss on workdays and social jetlag, similar to the participants of other studies [28], which might have resulted in a high prevalence of participants with excessive daytime sleepiness (ESS ≥  11).

The results of the multiple regression analyses showed a weak but significant relationship between sleep variables and some subcomponents of interoceptive awareness. The ESS score showed a significant relationship with “Noticing” and “Attention regulation” when the data of all participants were used for analysis, which means that participants with excessive daytime sleepiness tended to be aware of body sensation and had the ability to regulate distress by paying attention to body sensation. SLOSSweek, reflecting sleep loss, showed no relationship with MAIA scores, suggesting that sleep pressure *per se* is irrelevant to interoceptive awareness and that subjective interoceptive awareness might affect the sensitivity of sleep pressure. In other words, participants with higher interoception can detect increased sleep pressure and report their sleepiness correctly. This finding suggests that interoceptive awareness may explain the often-reported discrepancy between the subjective and objective measures of sleepiness in patients with obstructive sleep apnea [29] and those with chronic sleep loss [30]. However, there was also a negative relationship between ESS and the “Not distracting” subscale, which means the tendency not to ignore or distract oneself from bodily sensations of discomfort and pain. This indicates that participants with excessive daytime sleepiness tend to ignore or distract from their bodily sensations. Because this relationship was not significant only with the data of the participants without sleep problems, it may reflect the struggle of daytime workers who have severe sleepiness. However, the reason why the scores of “Not distracting” and other components have an inverse relationship with sleepiness is unclear. The score of “Not distracting” had a significant negative relationship with the score of “Attention regulation” in the original MAIA-J study [18]. Since this subscale score also showed negative relationships with the other components of MAIA in this study, it might reflect different aspects of interoceptive awareness from other components. Future longitudinal and experimental studies might be needed to further understand the relationship between the tendency to ignore the sensation of discomfort from their body and sleepiness.

When only the data of participants with neither excessive daytime sleepiness nor insomnia symptoms were included, the ESS score showed significant associations with “Emotional awareness”, “Body listening”, and “Trusting”. This result suggests that workers without sleep problems who recognize the connection between body sensations and emotional state listen to the body for insight and feel one’s body as safe and trustworthy tend to report their sleepiness higher independently from their sleep deficit, SJL, and chronotype. However, ESS scores were not significantly associated with “Noticing”, “Not distracting”, and “Attention regulation” subscales, in contrast to the results of the analysis using all participants’ data. While the discrepancy between the results of regression analyses with and without the data of participants with sleep problems might be partly explained by the low statistical power due to the limited sample size (n =  196) for exploratory analyses, it is possible that the relationship between interoceptive awareness and sensitivity for sleepiness are different in workers with and without sleep problems.

The AIS score was also negatively correlated with the “Not distracting” subscale when all the participants’ data were used for analysis, but the significant relationships were diminished when only the data of participants without sleep problems were used. Because chronic pain tends to be comorbid with insomnia [31] and people with insomnia tend to pay attention to internal and external environments [32], it is possible that people with insomnia symptoms also try to ignore bodily sensations in daily life. Arora et al. [5] reported a negative relationship between the “Not distracting” subscale and sleep quality measured by PSQI, which is consistent with our findings.

Variables related to dreaming also showed a significant relationship with MAIA subscales. In the present study, nightmare distress showed a significant positive relationship with “Emotional awareness” and a negative relationship with “Not distracting”. Additionally, there was a significant positive relationship between dream frequency and “Not distracting”. These results suggest that participants who struggle with nightmares have a higher awareness of the connection between body sensations and emotional states but try to distract themselves from signals from the body. This might suggest that experiences of nightmares increase the opportunity to realize the connectivity between body sensation and emotion, and that interoceptive awareness may explain individual differences in nightmare distress, as noted in previous studies [11,13]. However, there was no such relationship when the data of participants with excessive daytime sleepiness and insomnia symptoms were eliminated from the analysis. This suggests the necessity of exploring the difference in association of interoception with sleep variables including nightmares in the data of participants with and without sleep problems.

One limitation of this study is the reliability of responses. Data from more than 20% of the participants were eliminated from the analysis owing to unreliable answers for sleep onset and/or end time. As this was probably due to participants’ errors in judgment between AM and PM, it may be possible to correct the values according to certain criteria. However, in this study, to ensure the reproducibility of the results, we did not correct them, and data from participants who reported sleep patterns that were clearly inappropriate for full-time daytime workers were eliminated from all analyses. In addition, there might be a sampling bias in this study because this survey was conducted through a web research company.

The results of this study suggest that subjective daytime excessive sleepiness, in addition to nightmare distress and sleep quality, are associated with interoceptive awareness. This study is the first study pointing out the relationship between subjective daytime excessive sleepiness and interoceptive awareness. In this study, we targeted only daytime workers and the results showed that age was related to “Attention regulation”, suggesting a developmental change in interoceptive awareness. Further research should explore the relationship between sleep variables and interoceptive awareness in various age samples such as students and elderly adults. Additionally, we excluded part-time workers from the target population because their work schedule (e.g., work duration and start time of work) is thought to be much different from those of full-time workers, and it affects their sleep habits and sleep health. However, this exclusion criteria limits the generalizability of the results to all daytime workers. Further research targeting various type of workers, including part-time and shift-workers, should be conducted.

The participants in this study were limited to subjectively healthy people who were not currently visiting a hospital. However, the percentages of participants reporting excessive daytime sleepiness and insomnia symptoms were high. This might be caused by the research method with a web-based survey, and it is possible that non-diagnosed patients with sleep disorders such as obstructive sleep apnea and insomnia participated in this survey. The difference in the relationship between interoceptive awareness and sleep problems in objectively healthy controls and patients with sleep disorders should be explored in future studies. Moreover, a previous study suggests a difference in the interoception–sleep quality relationship between healthy controls and patients with psychological disorders [8]. Since the present study did not assess psychological conditions such as depression and anxiety, the relationship between interoception and sleepiness should be examined with consideration of interactions with mental health conditions. Further investigation of this relationship is expected to lead to a better understanding of sleep disorders and to elucidate individual differences in the accuracy of subjective assessments of sleepiness.

In this survey, interoception was subjectively assessed with MAIA, which is known to have low internal consistency, and its improved version (i.e., MAIA-2) was developed in 2018 [33]. Unfortunately, since there was no Japanese version of MAIA-2 at the time our survey was conducted, we used MAIA. Moreover, every variable in this study was based on subjective reports. Interoception can be objectively measured using heartbeat-evoked potentials [34] or the heartbeat count task [35]. Further research using various methods to measure interoception is expected to provide new insights into this research area.
